# Multimodal Visualization and Explainable Machine Learning–Driven Markers Enable Early Identification and Prognosis Prediction for Symptomatic Aortic Stenosis and Heart Failure With Preserved Ejection Fraction After Transcatheter Aortic Valve Replacement: Multicenter Cohort Study

**DOI:** 10.2196/70587

**Published:** 2025-05-01

**Authors:** Jun Wang, Jiajun Zhu, Hui Li, Shili Wu, Siyang Li, Zhuoya Yao, Tongjian Zhu, Bi Tang, Shengxing Tang, Jinjun Liu

**Affiliations:** 1 Department of Cardiology The First Affiliated Hospital of Bengbu Medical University Bengbu China; 2 Joint Research Center for Regional Diseases of IHM Bengbu Medical University Bengbu China; 3 Joint Research Center for Regional Diseases of IHM The First Affiliated Hospital of Bengbu Medical University Bengbu China; 4 Department of Cardiology First Affiliated Hospital of Xinjiang Medical University Urumchi China; 5 Department of Cardiology The People’s Hospital of Bozhou Bozhou China; 6 Department of Cardiology Xiangyang Central Hospital Xiangyang China; 7 Department of Cardiology First Affiliated Hospital of Wannan Medical College Wuhu China

**Keywords:** machine learning, interpretable models, heart failure with preserved ejection fraction, symptomatic aortic stenosis, transcatheter aortic valve replacement, major adverse cardiovascular and cerebrovascular events.

## Abstract

**Background:**

Currently, there is a paucity of literature addressing personalized risk stratification using multimodal data in patients with symptomatic aortic stenosis and heart failure with preserved ejection fraction (HFpEF) following transcatheter aortic valve replacement (TAVR).

**Objective:**

This study aimed to enhance the performance of risk assessment models in this patient population by developing a predictive model for adverse outcomes using various machine learning (ML) techniques.

**Methods:**

This multicenter cohort study included 326 patients diagnosed with severe AS and HFpEF who underwent TAVR between January 2017 and December 2023. Patients were allocated to training (n=195) and independent validation (n=131) sets based on hospital affiliation. A dual-phase feature selection process, combining least absolute shrinkage and selection operator logistic regression and the Boruta algorithm, was used to identify relevant variables from the multimodal dataset. A total of 5 ML model-decision trees, K-nearest neighbors, random forest, support vector machine, and extreme gradient boosting were used to construct a visualization and explainable predictive framework to elucidate model decision-making processes.

**Results:**

The primary features identified included age, N-terminal pro-brain natriuretic peptide, fasting blood glucose, triglyceride/high-density lipoprotein cholesterol ratio, triglyceride glucose index, triglyceride glucose-BMI index, atherogenic index of plasma index, and Apolipoprotein B. Among the 5 models, the support vector machine demonstrated the best predictive performance for major adverse cardiovascular and cerebrovascular events in patients with severe AS and HFpEF following TAVR, achieving an area under the curve of 0.756 (95% CI 0.631-0.881) in the independent validation set. The model exhibited good calibration and robust predictive power in both training and validation sets and demonstrated the highest net benefit in decision curve analysis compared to other models. To extract significant variables influencing the algorithm and ensure model appropriateness, we interpreted cohort and personalized model predictions using Shapley Additive Explanations values.

**Conclusions:**

Our ML-based multimodal model, incorporating 8 readily accessible predictors, demonstrated robust predictive capability for 12 months of major adverse cardiovascular and cerebrovascular events risk. This model can be used to identify high-risk individuals with AS and HFpEF following TAVR, potentially aiding in risk stratification and personalized treatment strategies.

## Introduction

The prevalence of heart failure with preserved ejection fraction (HFpEF), a common clinical syndrome, accounts for approximately 50% of all heart failure cases [1,2]. The incidence, mortality, and hospitalization rates associated with HFpEF are comparable to those of heart failure with reduced ejection fraction [3]. Furthermore, there is growing recognition that HFpEF may not be a single disease entity but rather a heterogeneous syndrome encompassing multiple distinct phenotypes [3]. This heterogeneity poses challenges for developing diagnostic, prognostic, and therapeutic strategies to improve outcomes in patients with HFpEF [3]. Current research is focused on identifying HFpEF subgroups characterized by metabolic dysfunction as potential pathophysiological mechanisms [3,4]. A comprehensive understanding of the underlying pathological processes and targeted therapeutic interventions may offer greater benefits than focusing solely on the final pathway of cardiac dysfunction. This recognition has led to a shift towards personalized medicine and subgroup-specific treatments.

Aortic stenosis (AS) and HFpEF are intricately connected, sharing common pathophysiological mechanisms that can exacerbate each other’s clinical manifestations [3,5,6]. Clinically, a substantial proportion of patients may present with both AS and HFpEF, often due to shared risk factors like advanced age, hypertension, obesity, and metabolic disturbances [3,5,6]. Transcatheter aortic valve replacement (TAVR) has emerged as a significant therapeutic intervention for severe AS in individuals aged 65 years and older [6]. However, despite its benefits, TAVR remains associated with a residual risk of adverse outcomes that significantly impact patient quality of life and survival [6]. Machine learning (ML) methodologies have been rapidly advancing, providing powerful tools for analyzing large and complex real-world datasets. These techniques can help identify patterns and correlations within data, enabling the identification of high-risk individuals [7-13]. Multicenter real-world datasets are particularly valuable for ML applications, as they often include diverse patient populations and extensive clinical data, reducing the risk of selection bias and missing data [7-10]. Our previous research has explored the application of ML to process multidimensional data, such as clinical texts, biomarkers, and imaging data, to identify risk factors for various cardiovascular diseases [8-13]. While these studies have demonstrated the potential of artificial intelligence in clinical practice [7], there is limited evidence regarding the use of ML models to predict outcomes in patients with concurrent AS and HFpEF following TAVR. To address this gap, we aimed to develop and validate a visualization and explainable prediction model using multimodal data for early, individualized risk stratification of these patients. By identifying high-risk individuals in a low-risk state, our model could potentially improve prognostication and inform novel therapeutic strategies for this vulnerable patient population.

## Methods

### Participants

**Figure 1:**
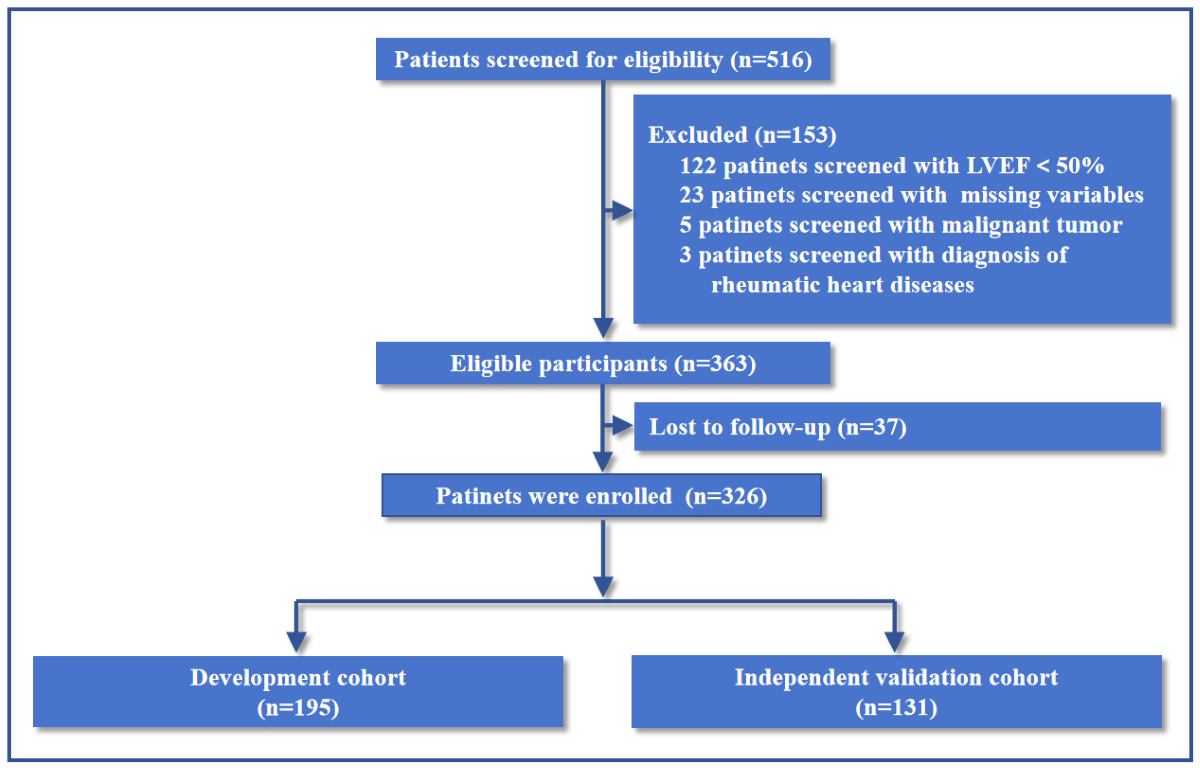
Flow diagram for patient selection. LVEF: left ventricular ejection fraction.

A multicenter, retrospective cohort study was conducted, analyzing consecutive older patients diagnosed with severe AS and HFpEF. All patients underwent TAVR at 4 tertiary care centers between January 2015 and December 2021. Comprehensive follow-up data were available for all participants included in the study. Patients were divided into a development set (n=195; datasets from The First Affiliated Hospital of Bengbu Medical University and the First Affiliated Hospital of Xinjiang Medical University) and an independent validation set (n=131; datasets from The First Affiliated Hospital of Wannan Medical College and Xiangyang Central Hospital). A team of heart specialists assessed patient suitability for TAVR, considering factors such as age, predicted lifespan, comorbidities, anatomical and procedural features, vascular access feasibility, surgical risks, bioprosthetic valve durability, and long-term outcomes. HFpEF diagnosis adhered to current guideline recommendations [14], including clinical symptoms and signs of heart failure, echocardiographic evidence of structural and/or functional cardiac abnormalities, and elevated N-terminal pro-brain natriuretic peptide (NT-proBNP) levels. TAVR procedures were performed according to standard clinical protocols. Detailed inclusion and exclusion criteria are provided in Table S1 in Multimedia Appendix 1, and the patient enrollment flowchart is depicted in Figure 1. The study was conducted in accordance with the Declaration of Helsinki.

#### Clinical Data

In our study, we meticulously documented the clinical characteristics, comorbidities, laboratory findings, and echocardiographic observations for all enrolled patients. To ensure data integrity and reliability, we exclusively used data derived from routine clinical examinations, mitigating potential issues associated with missing data (Multimedia Appendix 1). Before venous blood collection, individuals were instructed to fast for a minimum of 8 hours. Blood samples were collected on the morning following hospital admission and before TAVR. The triglyceride glucose (TyG) index was calculated as the natural logarithm of the product of fasting serum triglycerides (mg/dL) and fasting blood glucose (FBG; mg/dL), divided by 2 [15]. BMI was calculated as weight divided by height squared (kg/m²). Subsequently, the TyG index was refined by multiplying it with BMI, resulting in TyG-BMI indices [16]. The atherogenic index of plasma (AIP) was calculated as the logarithmically transformed ratio of triglyceride/high-density lipoprotein cholesterol (TG/HDL-C) expressed in mmol/L [17].

#### Clinical Endpoint

The study population was followed for a median follow-up duration of 12 months via outpatient visits and telephone interviews. The primary outcome of interest was the occurrence of major adverse cardiovascular and cerebrovascular events (MACCEs), encompassing cardiac death, revascularization, myocardial infarction, and stroke. All patients underwent regular follow-up assessments at 1, 3, 6, and 12 months post TAVR, with subsequent annual evaluations.

#### Feature Selection

In the feature selection process, we leveraged clinical expertise and current literature pertaining to the etiology, pathology, and treatment of HFpEF and AS. Adhering to predefined inclusion and exclusion criteria, we identified and incorporated 58 variables into our study. Recognizing the crucial role of variable selection in model performance, we used a 2-step feature selection process. First, we used the Boruta algorithm to analyze multiple high-dimensional and multivariable datasets. Subsequently, we applied the least absolute shrinkage and selection operator (LASSO) logistic regression to further refine the feature set.

By incorporating a penalty term during model estimation, the LASSO technique effectively reduces the regression coefficients of nonessential variables to 0, thereby facilitating variable selection by eliminating these variables from the model [18]. This approach significantly diminishes data dimensionality in high-dimensional data analysis and addresses issues of multicollinearity [18]. We implemented 10-fold cross-validation to optimize the lambda parameter in our LASSO regression, ensuring that our feature selection was robust against overfitting and selection bias.

The Boruta algorithm, a random forest-based feature selection method, identifies relevant features by contrasting them with irrelevant ones [19]. It iteratively trains multiple random forest models on bootstrapped samples, calculating feature importance using metrics like mean decrease impurity [19]. To establish a baseline, Boruta introduces shadow features—randomly permuted copies of the original features. By comparing the importance scores of original features to their shadow counterparts, the algorithm selects those that consistently outperform the shadows, thereby isolating the optimal set of relevant variables. This approach is effective for nonlinear, high-dimensional data, is robust to missing values and noise, and can be integrated with various classifiers or regressors to improve predictive performance, all without making assumptions about data distribution.

Ultimately, we used a rigorous 2-step feature selection methodology combining LASSO logistic regression and the Boruta algorithm. This approach effectively identified the most relevant predictors while minimizing the influence of confounding variables. This integrative approach aimed to enhance the model’s accuracy and generalizability while mitigating the risk of overfitting or incorporating irrelevant predictors.

#### Model Validation and Evaluation

Our study focused on the implementation and evaluation of 5 commonly used ML algorithms: decision tree (DT), k-nearest neighbors (KNN), random forest (RF), extreme gradient boosting (XGBoost), and support vector machine (SVM). The primary goal was to predict MACCEs in patients with HFpEF and AS who underwent TAVR. To assess the performance of these ML models, we used a comprehensive set of metrics, including the area under the receiver operating characteristic curve, area under the precision-recall curve, specificity, sensitivity, *F*_1_-score, recall, negative predictive value, positive predictive value, and Brier score. To evaluate the calibration of the models, we use calibration plots generated through 500-bootstrap resampling, which compared predicted probabilities of MACCEs to observed probabilities. In addition, decision curve analysis was conducted to assess the net clinical benefit of the model at varying threshold probabilities. Finally, feature ranking evaluation was performed to quantify the importance of individual features within the dataset and their contribution to predicting MACCEs.

#### Model Explainability

While the accurate interpretation of machine learning models remains challenging, the SHAP (Shapley Additive Explanations) method provides a robust solution by ranking input features and elucidating prediction outcomes, thereby addressing the “black-box” problem [20].

SHAP is a model interpretability technique rooted in game theory principles. It quantifies the contribution of individual feature values to a model’s prediction by assigning an explanatory score to each feature. SHAP values are derived from the concept of Shapley values, which involves permuting and integrating feature values to assess their influence on the model’s output. SHAP analysis enables the understanding of the underlying factors driving model predictions, providing intuitive explanations for the predicted outcomes. This not only deepens the understanding of the model’s decision-making logic and facilitates the identification of key risk factors but also provides a scientific basis for formulating individualized therapeutic strategies.

### Statistical Analysis

Statistical analysis of the study results was performed using SPSS (version 26; IBM Corp) and R software (version 4.2.2; R Foundation for Statistical Computing) To assess potential heterogeneity between development and validation cohorts, we conducted comprehensive statistical comparisons of all baseline characteristics. Categorical variables were characterized using descriptive statistics, including frequencies and proportions, while continuous variables were described using either medians and IQR or means and SDs. The nonparametric Mann-Whitney *U* test was used to compare continuous variables that exhibited nonnormal distributions. For the comparison of categorical variable distributions between the training and independent validation sets, either Fisher exact test or the chi square test was used. A significance level of *P*<.05 was considered for all statistical comparisons.

### Ethical Considerations

Ethical approval for this study was granted by the local research Ethics Committees of the First Affiliated Hospital of Bengbu Medical University (2024 KY065), the First Affiliated Hospital of Xinjiang Medical University (K202308-26), the First Affiliated Hospital of Wannan Medical College (2024 {130}), and Xiangyang Central Hospital (2024-169). Given the retrospective design of the study, the need to obtain informed consent from eligible patients was waived by the Ethics Committees.

## Results

### Patient Characteristics

A cohort of 326 patients diagnosed with HFpEF and AS was enrolled, using data collected from 4 independent hospital databases spanning the period from January 2017 to December 2023. Among all the participants included in the study, 195 individuals were assigned to the training group from 2 centers, while 131 individuals were allocated to the independent validation cohort from 2 other centers. This study adhered to the TRIPOD (Transparent Reporting of a multivariable prediction model for Individual Prognosis or Diagnosis) reporting guidelines (Table S2 in Multimedia Appendix 1).

Among the 326 patients, 43 (13.19%) experienced MACCEs. Specifically, within the training set, 21 (10.77%) patients had MACCEs, while in the validation set, 22 (16.79%) patients experienced MACCEs. Baseline characteristics were comparable across both sets, except for potassium ion, fibrinogen, white blood cell count, alanine aminotransferase, platelet distribution width, cystatin C, previous use of aspirin, beta-blockers, calcium channel blockers, angiotensin-converting enzyme inhibitors or angiotensin receptor blockers, platelet count, indirect bilirubin, sodium ion, and creatine kinase (*P*<.05; Table S3 in Multimedia Appendix 1).

#### Feature Selection

**Figure 2:**
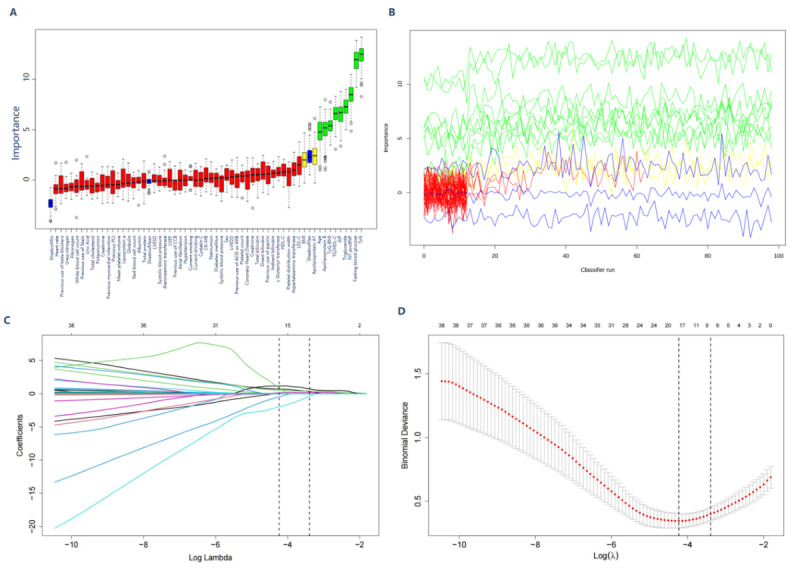
Selection of variables by using the Boruta algorithm for the purpose of selecting variables (A and B) and using Lasso regression for the purpose of selecting variables (C and D).

The Boruta algorithm assesses the impact of randomizing individual features on a classifier’s performance, thereby quantifying the unique information carried by each feature. Boruta feature selection identified age, BMI, NT-proBNP, fasting blood glucose, triglycerides, TG/HDL-C, TyG, TyG-BMI, AIP, apolipoprotein A1, and apolipoprotein B as potential predictors of MACCEs (Figures 2A and B).

To automatically select the most predictive features for MACCE risk, LASSO regression was used on the training dataset, as illustrated in Figures 2C and D. This technique minimizes the binomial deviance loss function by adjusting the regularization parameter, lambda (λ), which sets the coefficients of less important features to 0. At a shrinkage parameter (Lambda.min) of 0.01453175, 18 out of 48 features were selected: age, current smoking, heart rate, mean platelet volume, uric acid, NT-proBNP, coronary heart disease, diabetes mellitus, fasting blood glucose, TG/HDL-C, TyG, TyG-BMI, AIP, apolipoprotein B, aspartate aminotransferase, γ-Glutamyl Transferase, globulin, and creatine kinase.

**Figure 3:**
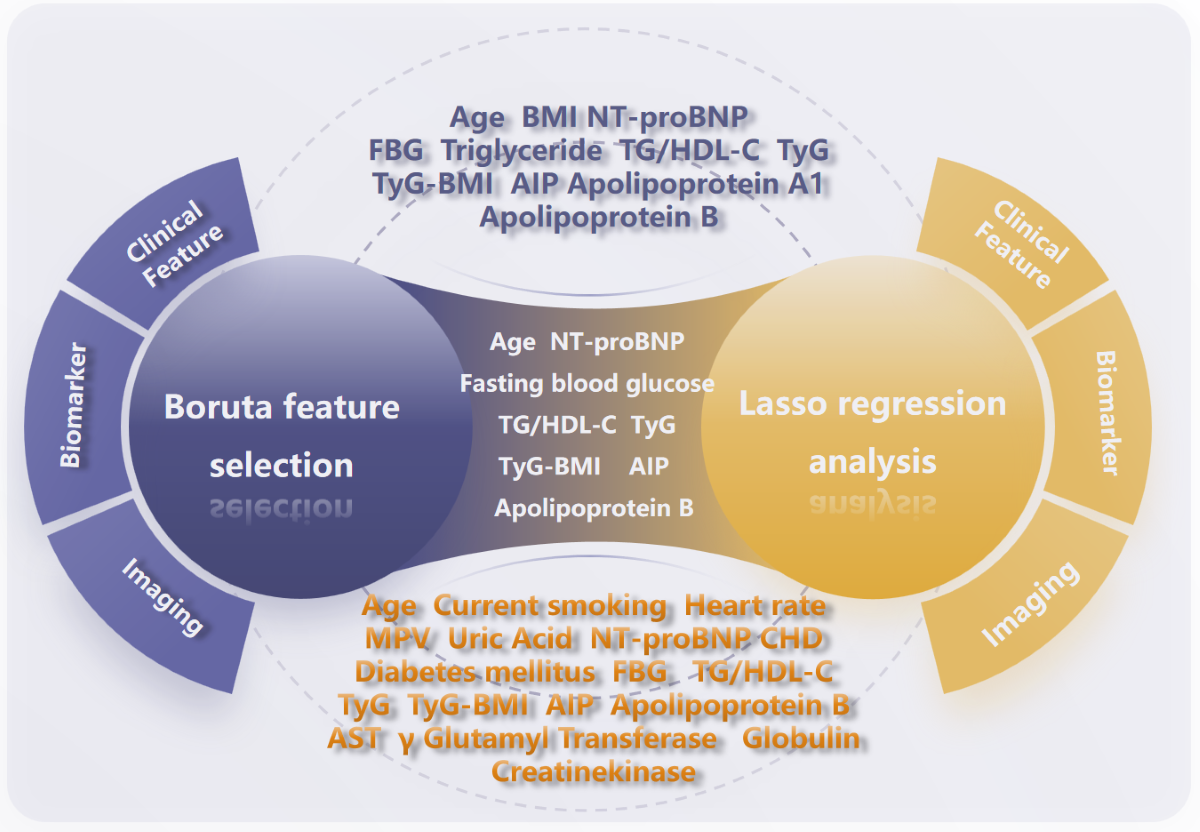
The Boruta algorithms and LASSO regression analysis identified a total of 8 variables. AIP: atherogenic index of plasma; AST: aspartate aminotransferase; CHD: coronary heart disease; FBG: fasting blood glucose; LASSO: least absolute shrinkage and selection operator; MPV: mean platelet volume; NT-proBNP: N-terminal pro-brain natriuretic peptide; TG: triglyceride; TG/HDL-C: triglyceride/high-density lipoprotein cholesterol; TyG: triglyceride glucose.

A dual-phase feature selection process was used, incorporating the Boruta algorithm and LASSO logistic regression. This combined approach identified 8 significant predictors of MACCE risk: age, NT-proBNP, fasting blood glucose, TG/HDL-C ratio, TyG index, TyG-BMI, AIP index, and apolipoprotein B (Figure 3).

#### Model Validation and Evaluation

We developed and optimized 5 widely recognized machine learning models: DT, KNN, RF, XGBoost, and SVM, with the goal of predicting the risk of MACCEs in patients with HFpEF and AS. These models were trained on the entire training set and subsequently evaluated on a separate validation set.

**Figure 4:**
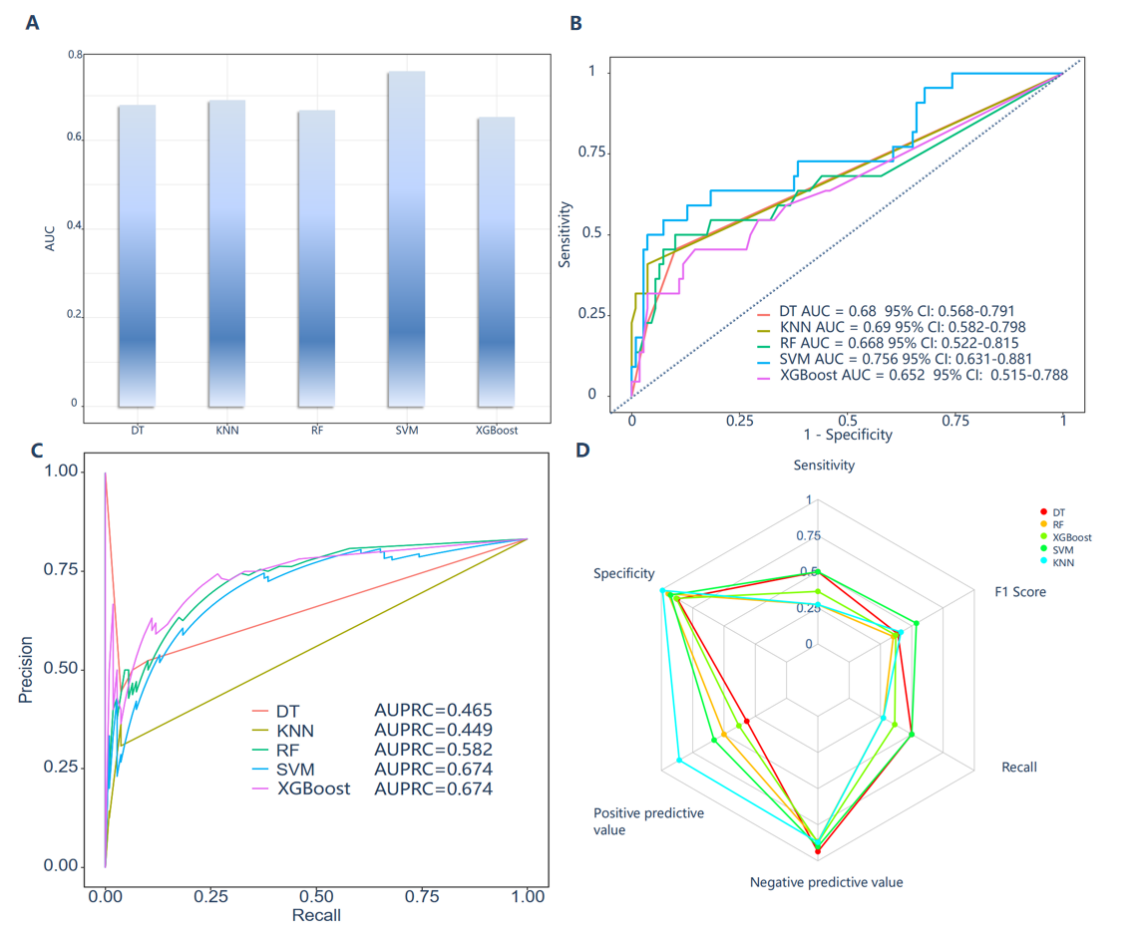
Area under the receiver operating characteristic curve (AUCs), area under the precision-recall curve (AUPRC), specificity, sensitivity, F1-score, recall, negative predictive value and positive predictive value for 5 machine learning in the validation set. DT: decision tree; KNN: k-nearest neighbors; RF: random forest; SVM: support vector machine; XGBoost: extreme gradient boosting.

Their performance was subsequently assessed using the training (Figures S1-S4 in Multimedia Appendix 1) and validation (Figures 4A-D) sets. To predict the risk of MACCEs, receiver operating characteristic curves were constructed for the 5 machine learning models. The SVM model demonstrated the highest area under the curve (AUC) at 0.756 (95% CI 0.631-0.881, and the lowest Brier score of 0.119 (95% CI 0.076-0.181) in validation sets, as illustrated in Figure 4 and Table S4 in Multimedia Appendix 1. The AUCs of the DT, KNN, RF, and XGBoost models were 0.68 (95% CI 0.568-0.791), 0.69 (95% CI 0.582-0.798), 0.668 (95% CI 0.522-0.815), and 0.652 (95% CI 0.515-0.788), with Brier scores of 0.149 (95% CI 0.098-0.218), 0.123 (95% CI 0.078-0.186), 0.125 (95% CI 0.081-0.178), and 0.129 (0.093-0.179), respectively (Figure 4B).

**Figure 5:**
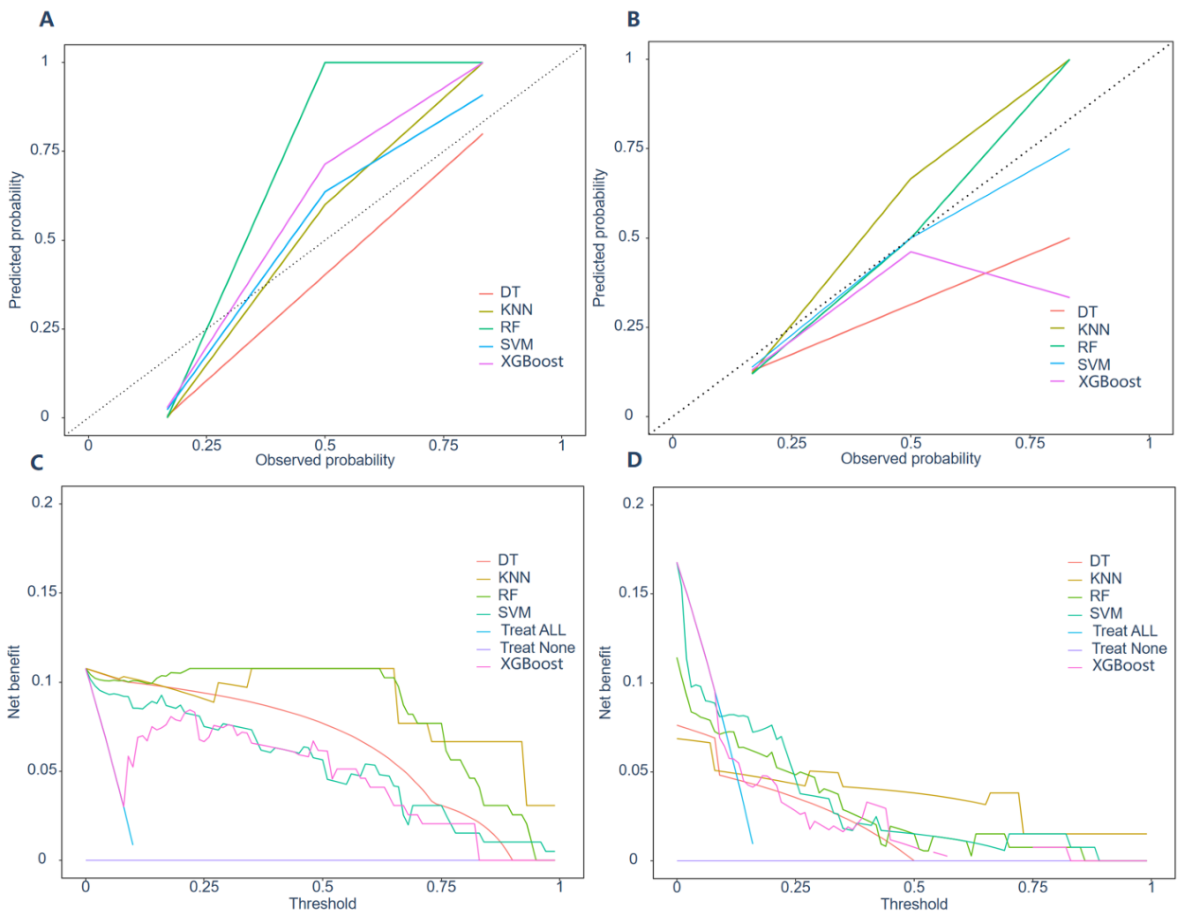
Calibration plots and decision curve analyses for 5 machine learning in training and validation sets. DT: decision tree; KNN: K-nearest neighbors; RF: random forest; SVM: support vector machine; XGBoost: extreme gradient boosting.

To comprehensively evaluate the model’s performance, metrics such as the area under the precision-recall curve, specificity, sensitivity, *F*_1_-score, recall, negative predictive value, positive predictive value, and Brier score were used, as depicted in Figure 4, Figures S1-S4 in Multimedia Appendix 1, and Table S3 in Multimedia Appendix 1. Figure 4 presents the optimal parameters identified for the 5 ML models used in predicting the risk of MACCEs using validation datasets. In addition, calibration curves indicated that the SVM model demonstrated superior calibration compared to the other 4 models (Figures 5A and B). As depicted in Figures 4 and 5, the SVM model was ultimately selected for predicting MACCEs in patients with AS and HFpEF due to its superior performance.

#### Clinical Utility of the Models

Decision curve analysis of the training set indicated that the SVM model outperformed DT, KNN, RF, XGBoost, and the strategies of treating all or none of the patients in terms of threshold probability and net benefit for predicting the risk of MACCEs (Figure 5C). Similarly, the SVM model consistently yielded higher net benefits in the validation set (Figure 5D).

The SVM model exhibited a high AUC of 0.978 (95% CI 0.958-0.999) on the training set, indicating strong discriminatory power. However, the model’s performance on the validation set was significantly weaker, where the AUC was substantially lower at 0.756 (95% CI 0.631-0.881), as illustrated in Figures S5 in Multimedia Appendix 1.

As shown in Figures S6 in Multimedia Appendix 1, a confusion matrix provides a comprehensive assessment of the model’s performance on both training and validation sets, detailing true positive, true negative, false positive, and false negative predictions. While the model demonstrated proficiency in predicting the negative class, it presented opportunities for improvement in detecting positive cases. Notably, the model achieved an impressive accuracy of approximately 94% and 85.4% on the training and validation sets, respectively.

### Model Interpretation

#### Overview

**Figure 6:**
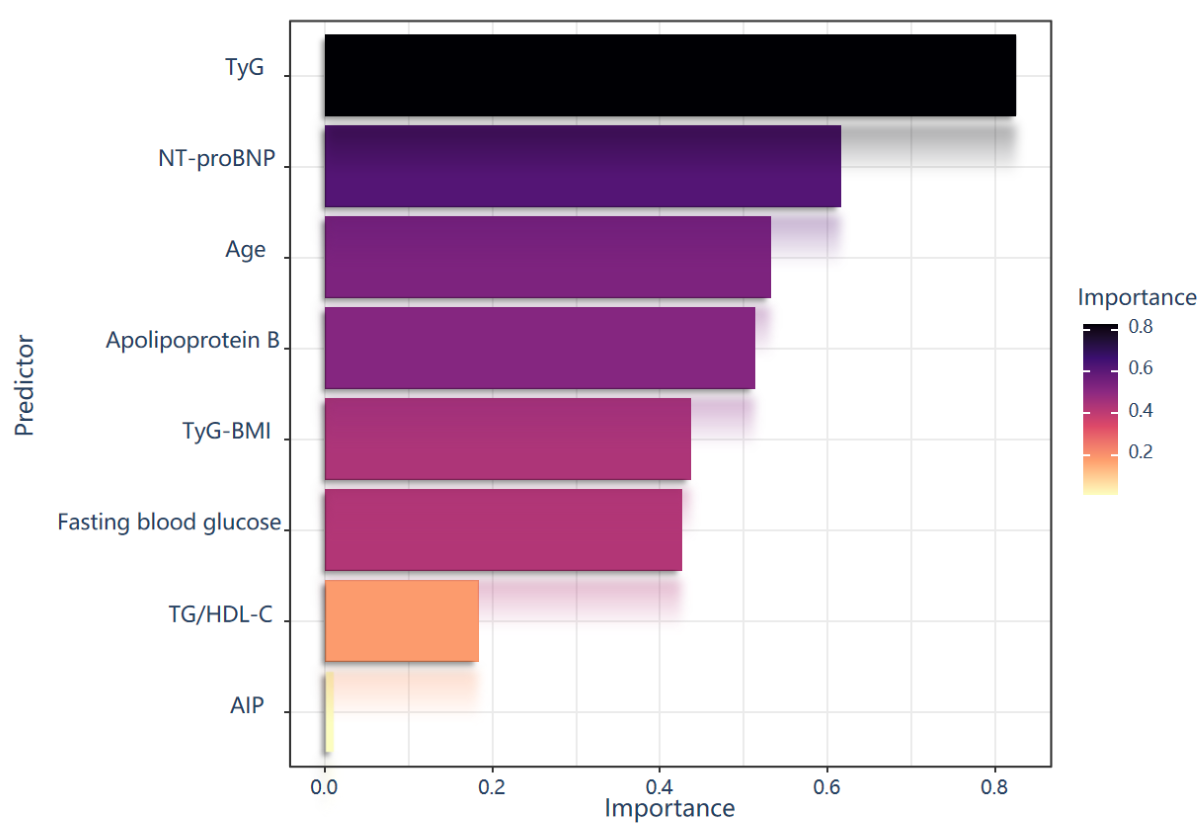
The variable importance of different predictors in a support vector machine model. Each bar represents a predictor variable, with the length of the bar indicating its relative importance in contributing to the model's predictions. AIP: atherogenic index of plasma; NT-proBNP: N-terminal pro-brain natriuretic peptide; TG/HDL-C: triglyceride/high-density lipoprotein cholesterol; TyG: triglyceride glucose.

**Figure 7:**
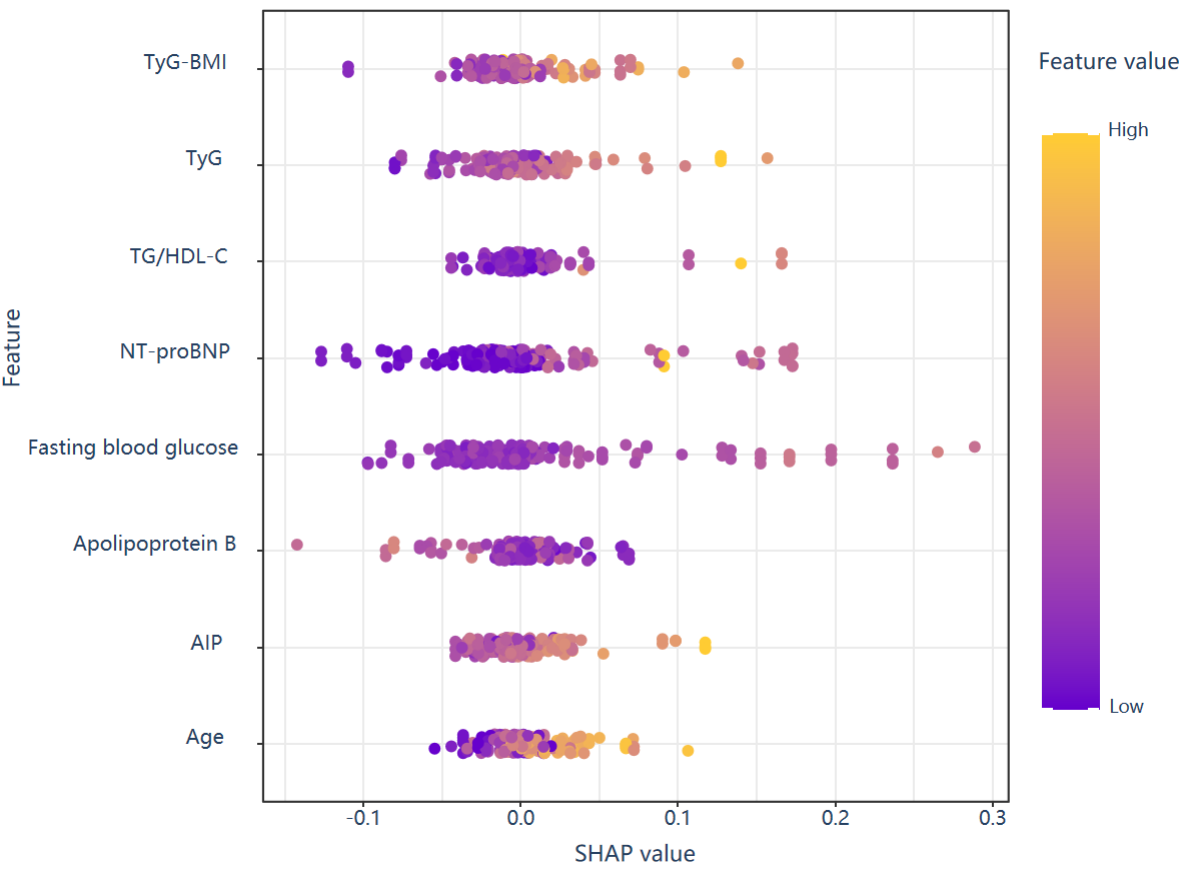
The relationship between feature values and their contributions to the model’s predictions, highlighting the most important factors influencing the major adverse cardiovascular and cerebrovascular events. AIP: atherogenic index of plasma; NT-proBNP: N-terminal pro-brain natriuretic peptide; SHAP: Shapley Additive Explanations; TG/HDL-C: triglyceride/high-density lipoprotein cholesterol; TyG: triglyceride glucose.

The SHAP method offers two distinct types of explanations: a global explanation at the feature level and a local explanation at the individual level. The global explanation elucidates the overall functionality of the model. As illustrated in the SHAP summary plots (Figures 6 and 7), the contributions of each feature to the model are assessed using average SHAP values and presented in descending order.

Figure 6 provides essential insights that inform subsequent analyses and feature selection for predictive modeling by identifying the predictors that significantly impact the performance of the SVM model. The features are organized in descending order based on their contribution to model performance. The TyG index exhibited the highest discriminative power, with an importance score of 0.82, and emerged as the most influential predictor, as evidenced by its notably longer bar compared to other variables. NT-proBNP was ranked second with an importance score of 0.62, followed by age (0.53), apolipoprotein B (0.51), TyG-BMI (0.43), and fasting blood glucose (0.42). The TG/HDL-C ratio and AIP demonstrated relatively modest contributions, with importance scores of 0.18 and 0.03, respectively.

To assess the significance of each predictive feature, we used the SHAP method. As illustrated in Figure 7, importance graphs rank these features in descending order of significance. SHAP values quantify the contribution of each feature to the final prediction outcome, enabling the interpretation of results for individual patients. Figure 7 represents SHAP values for individual observations across all predictive features. For each individual patient, a dot representing the SHAP value is plotted on the model for each specific feature, resulting in one dot per feature per patient. The color gradient of the dots indicates the actual feature values for each patient, with yellow signifying higher feature values and purple indicating lower feature values. The vertical stacking of the dots illustrates the density distribution. The horizontal distribution illustrates how feature values impact model predictions, with positive SHAP values indicating an increased risk of adverse outcomes. Metabolic parameters demonstrate distinct contributory patterns: elevated TyG index values (indicated by yellow points) consistently yield positive SHAP values, underscoring its efficacy as a robust risk predictor. Similarly, increased TyG-BMI values predominantly contribute positively, albeit with greater variability. Fasting blood glucose exhibits the most pronounced rightward extension, indicating a substantial increase in risk at higher levels. NT-proBNP displays a biphasic distribution, with both extremely low and high values exerting a significant impact on the model. Apolipoprotein B is associated with enhanced risk primarily at elevated concentrations. The TG/HDL-C ratio and AIP exhibit more diffuse patterns with bidirectional effects. Advanced age (also marked by yellow points) consistently results in positive SHAP values, reaffirming its established role as a nonmodifiable risk factor.

#### Facilitates Practical Application in Clinical Settings

The final prediction model was integrated into the web application to enhance its utility in clinical scenarios, as depicted in Figure S7 in Multimedia Appendix 1. Upon entering the actual values of the 8 features required by the model, the application will automatically estimate the 12-month risk of MACCEs for patients with AS and HFpEF following TAVR. Furthermore, a force plot for individual patients will be presented, illustrating the features contributing to the prediction of MACCEs. Specifically, the blue features on the right indicate factors reducing the likelihood of MACCEs, while the red features on the left increase the likelihood of MACCEs.

## Discussion

### Principal Findings

**Figure 8:**
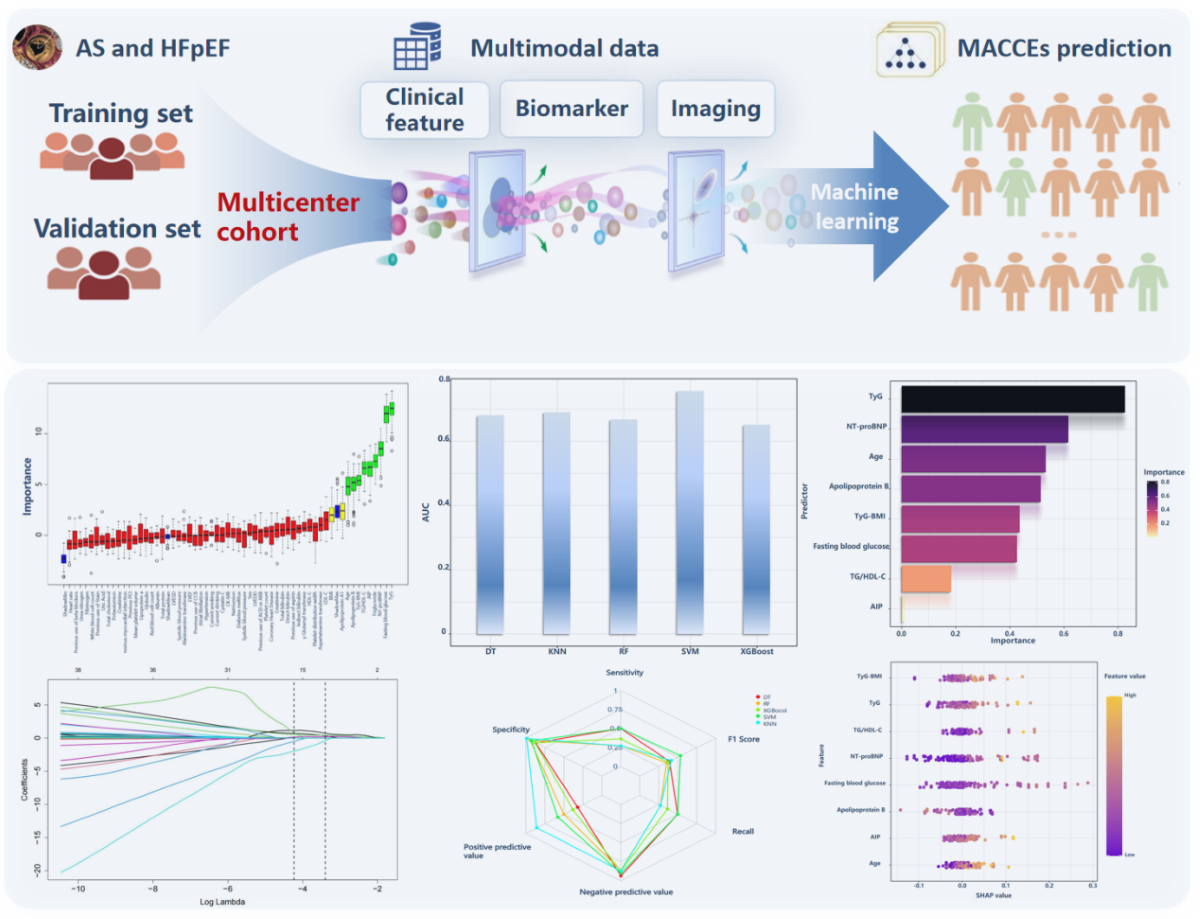
Central illustration. Multiple visualization and explainable machine learning driven integration reliable prognostic biomarkers that can identify “high-risk” patients with AS and HFpEF at an early stage, thereby facilitating the selection of suitable candidates for TAVR. AIP: atherogenic index of plasma; AS: aortic stenosis; DT: decision tree; KNN: k-nearest neighbors; MACCE: major adverse cardiovascular and cerebrovascular events; HFpEF: heart failure with preserved ejection fraction; NT-proBNP: N-terminal pro-brain natriuretic peptide; RF: random forest; SVM: support vector machine; TyG: triglyceride glucose; XGBoost: extreme gradient boosting.

Our study aimed to predict the 12-month risk of MACCEs in patients with AS and HFpEF following TAVR using a diverse range of ML models applied to multimodal data from a multicenter cohort (Figure 8 central illustration). To address the limitations in predicting and understanding the complex interplay of comorbidities associated with AS and HFpEF pathogenesis, we used interpretable ML techniques. Our models provide insights into the impact of various features on individual MACCE risk predictions, facilitating the development of hypotheses for early screening, prevention, and treatment strategies for patients with coexisting AS and HFpEF post TAVR. By leveraging multimodal data that encompasses the spectrum of metabolic heterogeneity and pathophysiological conditions observed in real-world clinical settings, our models accurately assess individual MACCE risk. We used a multimodal approach incorporating clinical text, biomarkers, and imaging data to comprehensively analyze the complexity of MACCE risk. We identified distinct high-risk subgroups for MACCEs in patients with AS and HFpEF, developed corresponding clinical association rules, and mapped differentially expressed metabolic biomarkers among MACCE cases within these subgroups. Furthermore, we dissected individual patient trajectories to identify risk biomarkers for MACCEs post TAVR in patients with AS and HFpEF, advancing the field of personalized preventive strategies.

Metabolic factors, such as dyslipidemia and insulin resistance, are frequently intertwined and serve as both risk factors for cardiovascular diseases and active contributors to the underlying pathophysiological processes of target organ damage [21]. These factors play a causal role in the initiation and progression of cardiovascular diseases [21]. Notably, metabolic disorders are increasingly recognized as significant pathological drivers of HFpEF [3,22], leading to the view of HFpEF as an abnormal metabolic syndrome encompassing not only cardiac dysfunction but also systemic metabolic derangements. Concurrently, metabolic disorders have emerged as important common risk factors for both AS and HFpEF [3,5,6]. Adipose degeneration precedes the onset of moderate AS and the development of significant hypertrophy [23]. Furthermore, initial metabolic alterations facilitate or accelerate left ventricular hypertrophy, subsequently leading to fibrosis and compensatory decompensation [23]. Therefore, it is essential to adopt a broader perspective that extends beyond the cardiac domain to encompass the comprehensive metabolic risk profile of the entire body [24,25]. Recognizing that metabolic disturbances can have a cascading effect on cardiac function, a holistic approach to cardiac assessment is crucial. This approach should involve the integration of multimodal data and the inclusion of noncardiac-specific metabolic biomarkers to enable a thorough evaluation of patients with concurrent AS and HFpEF following TAVR.

In this study, we conducted a retrospective multicenter cohort analysis to apply a combination of multiple ML techniques to facilitate the early and accurate prediction of the risk of MACCEs in patients with AS and HFpEF who underwent TAVR. Our study incorporated data from 4 independent hospitals, enhancing the geographic and demographic diversity of our cohort and improving external validity. This predictive model quantified the predictive impact of both nonmodifiable risk factors, such as age, and baseline metabolic characteristics, including FBG levels, TG/HDL-C ratio, TyG index, TyG-BMI, AIP, and apolipoprotein B, in addition to clinical biomarkers such as NT-proBNP. A study by Chen et al [26] involving 1569 patients with AS who underwent TAVR over a follow-up period of 1.09 years revealed a positive linear relationship between insulin resistance, as assessed by the TyG index, and all-cause mortality, cardiovascular mortality, and major adverse cardiovascular events in this patient population [26]. In addition, the potential of FBG levels, TyG-BMI, AIP, TG/HDL-C, and apolipoprotein B in preventing artery stiffness and multiple cardiovascular events has been increasingly recognized [16,17,27-29]. Furthermore, NT-proBNP has been established as a significant predictor of cardiovascular events and heart failure in patients with AS, emphasizing the importance of comprehensive risk assessment and management strategies that incorporate these metabolic and clinical biomarkers to optimize patient outcomes [30]. Age, as a well-established risk factor, correlates with increased risks, highlighting the need for tailored interventions that consider the composite impact of these biomarkers and age on prognosis [31]. This underscores the necessity for routine screening and monitoring of these biomarkers, particularly in older individuals, to mitigate the potential risks of cardiovascular diseases and improve overall patient care. The incorporation of these biomarkers significantly improved the predictive accuracy and reliability of individual-level predictions, surpassing the performance achieved solely by relying on established risk factors (such as age and NT-proBNP). Furthermore, our model exhibited outstanding performance on both the training and independent validation datasets, indicating robust predictive capability and stability across diverse datasets. This suggests that metabolic dysfunction augments proinflammatory signaling, potentially accelerating cardiac fibrosis within the pathophysiology of HFpEF, which persists even following valve replacement. Furthermore, insulin resistance contributes to vascular stiffening, and when coupled with ventricular stiffness in HFpEF, it creates unfavorable loading conditions that may impede functional improvement despite successful valve replacement.

Notably, the differentiation of these risk biomarkers at the individual level has historically been hindered by the varying effects of different risk biomarkers on individuals. Currently, SHAP is used to quantify the feature contributions in predicting outcomes, facilitating the comprehension of the model’s predictive rationale [32]. This insight could potentially revolutionize patient care by offering a tailored and targeted approach that considers the unique interplay of each individual’s biomarkers [32]. In our study, a key strength lies in the application of explainable artificial intelligence techniques, specifically SHAP estimation, to assess the impact of distinct risk biomarkers within the framework of all integrated variables at the individual level. Furthermore, all of the predictors included in our model are easily obtainable in clinical practice, although some may require computational transformation. To enable the integration of our predictive model into clinical practice, we developed an extensive web-based application incorporating the SVM algorithm along with its interpretability features. This platform was designed with a responsive front end to ensure compatibility with a range of devices frequently used in clinical environments. This is beneficial to all levels of hospitals to address shortcomings and improve personalized treatment. Important, that the coexistence of AS and HFpEF markedly enhances the complexity of the disease, necessitating evaluation that extends beyond mere procedural success. Our model addresses this clinical challenge by incorporating metabolic parameters, particularly the TyG index, to assess preprocedural risk profiles. In the context of the coexistence of AS and HFpEF, while procedural success is necessary, it alone does not guarantee favorable long-term outcomes; the patient’s response to the intervention is equally critical. Our model provides significant value in this context by aiding clinicians in identifying patients who are likely to experience meaningful clinical benefits from TAVR, despite the presence of complex comorbidities. In addition, our model also prompts the crucial inquiry into tailored interventions, augmenting the precision in addressing cardio-metabolic disorders. The metabolic characteristics play a critical role in distinguishing the high risk of MACCEs among patients with AS and HFpEF after TAVR, thereby establishing a robust foundation for developing an individualized residual metabolic risk stratification model. Furthermore, this contributes to our understanding of potential windows for preventative interventions, identifying risk biomarkers when patients are at high risk. The assessment of metabolic status offers a critical insight into the patient’s physiological reserve and adaptive capacity, which are determinants of postprocedural recovery and long-term outcomes. In clinical practice, the model could be integrated into Heart Team discussions to stratify patients not only by surgical risk but also by potential functional improvement, based on their metabolic profile. Future validation studies might prospectively assess the impact of preprocedural metabolic optimization on outcomes in complex cases, with a particular emphasis on those with concomitant AS and HFpEF. This strategy signifies a significant advancement in the pursuit of personalized intervention timing and patient selection within the management of structural heart disease.

### Study Limitations

First, it is important to note that all samples used in this study were retrospective. Consequently, future validation of our model should be conducted within a prospective, multicenter cohort. Second, medical practices and standards are subject to evolution over time, and consequently, our model may not accurately reflect contemporary patient populations or current medical techniques. Third, due to the single-time-point nature of our study, the predictive power of our model might be limited, as it could not capture the dynamic changes of these risk factors over time. Nonetheless, our findings serve as a stepping stone for further research in this field, aiming to refine the prediction model and enhance the clinical application of biomarkers in preventing MACCEs.

### Conclusions

We conducted a multicenter cohort study using diverse ML techniques to prognosticate the risk of MACCEs in patients with AS and HFpEF after TAVR. The model incorporated age and an extensive array of metabolic biomarkers. The application of interpretable ML at the individual patient level enabled the early prediction of 1-year MACCEs through personalized modifiable risk biomarkers, thereby providing opportunities for tailored preventive strategies. Our incorporation of metabolic features in predicting MACCE-specific risk biomarkers highlights the predictive significance of the underlying biology associated with concurrent AS and HFpEF.

## Appendix

Multimedia Appendix 1 Additional material.

## Abbreviations

AIP: atherogenic index of plasma
AS: aortic stenosis
AUC: area under the curve
DT: decision tree
FBG: fasting blood glucose
HFpEF: heart failure with preserved ejection fraction
KNN: k-nearest neighbors
LASSO: least absolute shrinkage and selection operator
MACCE: major adverse cardiovascular and cerebrovascular events
ML: machine learning
NT-proBNP: N-terminal pro-brain natriuretic peptide
RF: random forest
SHAP: Shapley Additive Explanations
SVM: support vector machine
TAVR: transcatheter aortic valve replacement
TG/HDL-C: triglyceride/high-density lipoprotein cholesterol
TRIPOD: Transparent Reporting of a multivariable prediction model for Individual Prognosis or Diagnosis
TyG: triglyceride glucose
XGBoost: extreme gradient boosting

## Data Availability

Individual participant data that underlie the results reported in this article, after deidentification, can be obtained from the corresponding author upon reasonable request.
