# Supplementary material for: Multimodal Visualization and Explainable Machine Learning–Driven Markers Enable Early Identification and Prognosis Prediction for Symptomatic Aortic Stenosis and Heart Failure With Preserved Ejection Fraction After Transcatheter Aortic Valve Replacement: Multicenter Cohort Study
Source: J Med Internet Res. 2025 May 1;27:e70587. doi: 10.2196/70587 (PMC12082054; doi:10.2196/70587)
Supplement: Multimedia Appendix 1 [file jmir_v27i1e70587_app1.pdf]

Table S1. Inclusion and exclusion criteria.

**Inclusion criteria**
Aortic stenosis
Heart failure with preserved ejection fraction
Patients had successful TAVR implantation
Follow-up was completed
**Exclusion criteria**
Acute myocardial infarction
Acute cardiac failure
Implantation of pacemaker
Depressed left ventricular systolic function (ejection fraction <50%)
Malignant tumor
Dilated cardiomyopathy
Rheumatic heart disease
Myocarditis or cardiomyopathy
Infectious or severe liver or kidney disease
Missing variables
Poor compliance to treatment

Table S2. Checklist

Section/Topic Item		Development / evaluation[1]	Checklist item	Page
**TITLE**				
Title	1	D;E	Identify the study as developing or evaluating the performance of a multivariable prediction model, the target population, and the outcome to be predicted	1
**ABSTRACT**				
Abstract	2	D;E	See TRIPOD+AI for Abstracts checklist	3-4
**INTRODUCTION**				
Background	3a	D;E	Explain the healthcare context (including whether diagnostic or prognostic) and rationale for developing or evaluating the prediction model, including references to existing models	3
	3b	D;E	Describe the target population and the intended purpose of the prediction model in the context of the care pathway, including its intended users (e.g., healthcare professionals, patients, public)	3
	3c	D;E	Describe any known health inequalities between sociodemographic groups	3
Objectives	4	D;E	Specify the study objectives, including whether the study describes the development or validation of a prediction model (or both)	3
**METHODS**				
Data	5a	D;E	Describe the sources of data separately for the development and evaluation datasets (e.g., randomised trial, cohort, routine care or registry data), the rationale for using these data, and representativeness of the data	4
	5b	D;E	Specify the dates of the collected participant data, including start and end of participant accrual; and, if applicable, end of follow-up	4
Participants	6a	D;E	Specify key elements of the study setting (e.g., primary care, secondary care, general population) including the number and location of centres	4
	6b	D;E	Describe the eligibility criteria for study participants	4
	6c	D;E	Give details of any treatments received, and how they were handled during model development or evaluation, if relevant	4
Data preparation	7	D;E	Describe any data pre-processing and quality checking, including whether this was similar across relevant sociodemographic groups	5
Outcome	8a	D;E	Clearly define the outcome that is being predicted and the time horizon, including how and when assessed, the rationale for choosing this outcome, and whether the method of outcome assessment is consistent across sociodemographic groups	5
	8b	D;E	If outcome assessment requires subjective interpretation, describe the qualifications and demographic characteristics of the outcome assessors	5

	8c	D;E	Report any actions to blind assessment of the outcome to be predicted	5
Predictors	9a	D	Describe the choice of initial predictors (e.g., literature, previous models, all available predictors) and any pre-selection of predictors before model building	6
	9b	D;E	Clearly define all predictors, including how and when they were measured (and any actions to blind assessment of predictors for the outcome and other predictors)	6
	9c	D;E	If predictor measurement requires subjective interpretation, describe the qualifications and demographic characteristics of the predictor assessors	-
Sample size	10	D;E	Explain how the study size was arrived at (separately for development and evaluation), and justify that the study size was sufficient to answer the research question. Include details of any sample size calculation	-
Missing data	11	D;E	Describe how missing data were handled. Provide reasons for omitting any data	10
Analytical methods	12 a	D	Describe how the data were used (e.g., for development and evaluation of model performance) in the analysis, including whether the data were partitioned, considering any sample size requirements	6
	12 b	D	Depending on the type of model, describe how predictors were handled in the analyses (functional form, rescaling, transformation, or any standardisation).	6
	12 c	D	Specify the type of model, rationale[2], all model-building steps, including any hyperparameter tuning, and method for internal validation	6
	12 d	D;E	Describe if and how any heterogeneity in estimates of model parameter values and model performance was handled and quantified across clusters (e.g., hospitals, countries). See TRIPOD-Cluster for additional considerations[3]	6
	12 e	D;E	Specify all measures and plots used (and their rationale) to evaluate model performance (e.g., discrimination, calibration, clinical utility) and, if relevant, to compare multiple models	6
	12 f	E	Describe any model updating (e.g., recalibration) arising from the model evaluation, either overall or for particular sociodemographic groups or settings	6
	12 g	E	For model evaluation, describe how the model predictions were calculated (e.g., formula, code, object, application programming interface)	6
Class imbalance	13	D;E	If class imbalance methods were used, state why and how this was done, and any subsequent methods to recalibrate the model or the model predictions	7
Fairness	14	D;E	Describe any approaches that were used to address model fairness and their rationale	7
Model output	15	D	Specify the output of the prediction model (e.g., probabilities, classification). Provide details and rationale for any classification and how the thresholds were identified	7

Training versus evaluation	16	D;E	Identify any differences between the development and evaluation data in healthcare setting, eligibility criteria, outcome, and predictors	8
Ethical approval	17	D;E	Name the institutional research board or ethics committee that approved the study and describe the participant-informed consent or the ethics committee waiver of informed consent	8
**OPEN SCIENCE**				
Funding	18a	D;E	Give the source of funding and the role of the funders for the present study	18
Conflicts of interest	18b	D;E	Declare any conflicts of interest and financial disclosures for all authors	18
Protocol	18c	D;E	Indicate where the study protocol can be accessed or state that a protocol was not prepared	-
Registration	18d	D;E	Provide registration information for the study, including register name and registration number, or state that the study was not registered	-
Data sharing	18e	D;E	Provide details of the availability of the study data	18
Code sharing	18f	D;E	Provide details of the availability of the analytical code[4]	-
**PATIENT & PUBLIC INVOLVEMENT**				
Patient & Public Involvement	19	D;E	Provide details of any patient and public involvement during the design, conduct, reporting, interpretation, or dissemination of the study or state no involvement.	Supplementary Table 1
**RESULTS**				
Participants	20a	D;E	Describe the flow of participants through the study, including the number of participants with and without the outcome and, if applicable, a summary of the follow-up time. A diagram may be helpful.	7-8
	20b	D;E	Report the characteristics overall and, where applicable, for each data source or setting, including the key dates, key predictors (including demographics), treatments received, sample size, number of outcome events, follow-up time, and amount of missing data. A table may be helpful. Report any differences across key demographic groups.	7-8
	20c	E	For model evaluation, show a comparison with the development data of the distribution of important predictors (demographics, predictors, and outcome).	8-14
Model development	21	D;E	Specify the number of participants and outcome events in each analysis (e.g., for model development, hyperparameter tuning, model evaluation)	8-14
Model specification	22	D	Provide details of the full prediction model (e.g., formula, code, object, application programming interface) to allow predictions in new individuals and to enable third-party evaluation and implementation, including any restrictions to access or	8-14

			re-use (e.g., freely available, proprietary)[5]	
Model performance	23 a	D;E	Report model performance estimates with confidence intervals, including for any key subgroups (e.g., sociodemographic). Consider plots to aid presentation.	8-14
	23 b	D;E	If examined, report results of any heterogeneity in model performance across clusters. See TRIPOD Cluster for additional details[3].	-
Model updating	24	E	Report the results from any model updating, including the updated model and subsequent performance	-
**DISCUSSION**				
Interpretation	25	D;E	Give an overall interpretation of the main results, including issues of fairness in the context of the objectives and previous studies	14-17
Limitations	26	D;E	Discuss any limitations of the study (such as a non-representative sample, sample size, overfitting, missing data) and their effects on any biases, statistical uncertainty, and generalizability	14-17
Usability of the model in the context of current care	27 a	D	Describe how poor quality or unavailable input data (e.g., predictor values) should be assessed and handled when implementing the prediction model	-
	27 b	D	Specify whether users will be required to interact in the handling of the input data or use of the model, and what level of expertise is required of users	-
	27 c	D;E	Discuss any next steps for future research, with a specific view to applicability and generalizability of the model	-

From: Collins GS, Moons KGM, Dhiman P, et al. *BMJ* 2024;385:e078378.
doi:10.1136/bmj-2023-078378.

Table S3. Baseline patient characteristics and clinical outcome in the training and independent validation sets.*

Characteristic or outcome	All patients (n=326)	Training set (n=195)	Independent validation set (n=131)	P value
Sex (male) , n (%)	184 (56.44)	105 (53.85)	79 (60.31)	.30
Age (years)	75[70;80]	75 [70;81]	75[70.5;79]	.87
Atrial fibrillation, n (%)	55 (16.87)	31 (15.9)	24 (18.32)	.67
Hypertension, n (%)	153 (46.93)	99 (50.77)	54 (41.22)	.11
Coronary heart disease, n (%)	103 (31.6)	63 (32.31)	40 (30.53)	.83
Diabetes mellitus, n (%)	34 (10.43)	18 (9.23)	16 (12.21)	.50
Current smoking, n (%)	47 (14.42)	24 (12.31)	23 (17.56)	.25
Current drinking, n (%)	35 (10.74)	18 (9.23)	17 (12.98)	.37
Previous PCIa, n (%)	28 (8.59)	13 (6.67)	15 (11.45)	.19
Previous myocardial infarction, n (%)	13 (3.99)	9 (4.62)	4 (3.05)	.68
Previous use of aspirin, n (%)	39 (11.96)	37 (18.97)	2 (1.53)	<.001
Previous use of Stains, n (%)	13 (3.99)	9 (4.62)	4 (3.05)	.68
Previous use of beta blockers, n (%)	30 (9.2)	30 (15.38)	0	<.001
Previous use of CCBb, n (%)	48 (14.72)	48 (24.62)	0	<.001
Previous use of ACEIc or ARBd, n (%)	42 (12.88)	40 (20.51)	2 (1.53)	<.001
Systolic blood pressure, mmHg	128.5 [111-138]	128 [111-138]	130 [110.5-139]	.96
Diastolic blood pressure, mmHg	70[61-770]	70[60-78]	70[62-76]	.77
Heart rate, Beats per minute	75 [68-82]	75[67-83]	74 [68-82]	.66
BMI, kg/m2	25 [23.01-26.25]	25 [22.89-26.59]	25 [23.85-25.92]	.75
White blood cell count, 10^9/L	5.42 [4.6-6.76]	5.63 [4.79-6.9]	5.07 [4.4-6.22]	.01
Red blood cell count, 1012/L	4.14 [3.8-4.42]	4.11 [3.69-4.45]	4.22 [3.86-4.38]	.42
Platelet count, 10^9/L	154 [117-197.75]	164 [129.5-208.5]	132 [111-182.5]	<.001
Mean platelet volume, fL	11 [10.3-11.7]	11[10.3-11.65]	11.1 [10.4-11.8]	.27
Platelet distribution width	16.1 [14.4-16.4]	16.2 [15.3-16.4]	16 [13.9-16.4]	.03
Urea nitrogen, mmol/L	6.74 [5.72-8.11]	6.74 [5.56-8.32]	6.71 [6.09-7.92]	.65
Creatinine, umol/L	73.3 [63-83]	71 [60.92-87.05]	74 [68.5-80]	.26
Uric Acid, umol/L	324 [289.5-405.55]	338 [276.3-410.3]	316 [293.9-386.5]	.29
Total cholesterol, mmol/L	3.14 [2.74-3.96]	3.26	3.04 [2.8-3.64]	.35

		[2.64-4.13]		
NT-proBNPe, ng/mL	633.85 [313.35-1400.75]	712 [263-1711.92]	596.2 [381.55-999.4]	.74
Fasting blood glucose, mmol/L	4.84 [4.59-5.95]	4.98 [4.59-6.03]	4.78 [4.57-5.7]	.15
Triglyceride, mmol/L	1.02 [0.76-1.37]	1 [0.74-1.46]	1.02 [0.84-1.29]	.87
HDL-Cf, mmol/L	1 [0.87-1.21]	1.01 [0.87-1.27]	0.99 [0.89-1.17]	.30
LDL-Cg, mmol/L	2.3 [1.65-2.48]	2.27 [1.58-2.54]	2.34 [1.77-2.46]	.29
TG/HDL-Ch	1.04 [0.67-1.34]	1 [0.63-1.45]	1.06 [0.73-1.31]	.65
TyGi	8.29 [8.03-8.75]	8.3 [7.96-8.82]	8.28 [8.06-8.68]	.9
TyG-BMIj	206.16 [191.8-222.37]	206.11 [189.55-227.33]	206.28 [194.71-220.54]	.95
Atherogenic index of plasma	0.02 [–0.18 to 0.13]	0 [–0.2 to 0.16]	0.03 [–0.13 to 0.12]	.65
Apolipoprotein A1, mmol/L	1.21 [0.97-1.31]	1.2 [0.99-1.31]	1.21 [0.94-1.3]	.28
Apolipoprotein B, mmol/L	0.48 [0.47;0.7]	0.48 [0.47;0.71]	0.48 [0.47;0.69]	.62
Lipoprotein a, mg/L	106.4 [59.7-209.22]	133 [62.8-191.5]	95.4 [56 -23]	.34
Aspartateamino transferase, U/L	21 [17-28]	21 [17-27.5]	20 [17-28.5]	.67
Alanineamino transferase, U/L	17 [12-23]	16 [12-23]	19 [14.5-23]	.03
Gamma-glutamyl transferase, U/L	20 [14-29]	21 [14-29]	19 [13.5-29]	.38
Total bilirubin, μmol/L	12.1 [10-18.34]	13.12 [9.8-19.32]	11.45 [10.36-15.55]	.05
Direct bilirubin, μmol/L	4.45 [2.7-6.7]	4.3 [2.7-6.27]	4.9 [2.75-7.25]	.09
Indirect bilirubin, μmol/L	8.25 [5.3-12.07]	9.1 [6.16-13.15]	7.1 [4.8-9.8]	<.001
Total protein, g/L	65.3 [62.7-67.5]	65.3 [62.4-67.4]	65.3 [63.05-67.5]	.69
Albumin, g/L	37.35 [35.23-40.6]	37.5 [35.1-40.45]	37.2 [35.6-41]	.52
Globulin, g/L	26.3 [23.92-29.2]	26.3 [23.5-29]	26.4 [24.45-29.3]	.53
Potassium ion, mmol/L	3.9 [3.56-4.23]	3.95 [3.71-4.24]	3.68 [3.47-4.16]	.001
Sodium ion, mmol/L	141 [139-143.67]	142 [140-144.3]	140 [138.1-142]	<.001
Creatinekinase, U/L	47 [29-71]	52 [32-74]	35 [29-64]	<.001
Creatine kinase-MB, U/L	11 [9-13.8]	12 [9-14]	11 [9-13.04]	.61
Cystatin C mg/L	1.19 [1-1.5]	1.16	1.22 [1.06-1.5]	.03

		[0.96-1.49]		
Fibrinogen, g/L	2.72 [2.46-3.03]	2.75 [2.46-3.37]	2.67 [2.46-2.85]	.006
LVEDDj, mm	50 [49-53]	50 [49-55]	50 [47.5-52]	.09
LVESDk, mm	39 [35.25-42]	39 [35-41]	39 [36-42.5]	.42
LVEFl, %	55 [51;59]	54 [51;59]	55 [51;59]	.41
MACCEs, n (%)	43 (13.19)	21 (10.77)	22 (16.79)	.16
Cardiac death, n (%)	29 (8.9)	14 (7.18)	15 (11.45)	.26
Revascularization, n (%)	7 (2.15)	3 (1.45)	4 (3.05)	.45
Myocardial infarction, n (%)	3 (0.92)	1 (0.51)	2 (1.53)	.57
Stroke, n (%)	9 (2.76)	3 (1.54)	6 (4.58)	.17

* Mean ± SD, for normally distributed data. Median [Interquartile Range (IQR)], for non-normally distributed data. Categorical variables are presented as frequencies and percentages: n (%).

[a]PCI:, Percutaneous coronary intervention

[b]CCB: Calcium Calcium Entry Blockers

[c]ACEI: Angiotensin-Converting Enzyme Inhibitors

[d]ARB: Angiotensin Receptor Blockers

[e]NT-proBNP: N-terminal pro-brain natriuretic peptide

[f]HDL-C: high-density lipoprotein cholesterol

[g]LDL-C: low-density lipoprotein cholesterol

[h]TG/HDL-C: triglyceride/high-density lipoprotein cholesterol

[i]TyG: triglyceride glucose

[j]LVEDD: left ventricular end-diastolic diameter

[k]LVESD: left ventricular end-systolic diameter

[l]LVEF: left ventricular ejection fraction

Table S4. Brier score of training and validation sets.

	Training set		Validation set	
	**Brier Score**	**95% CI**	**Brier Score**	**95% CI**
XGboost	0.0476	0.0357-0.0685	0.1285	0.0929-0.1796
SVM	0.0385	0.0249-0.0609	0.1191	0.0760-0.1812
RF	0.0078	0.0052-0.0118	0.1251	0.0809-0.1784
KNN	0.0095	0.0061-0.0148	0.1230	0.0783-0.1859
DT	0.0244	0.0120-0.0501	0.1496	0.0978-0.2181

Figure S1. AUROCs for models in training set.

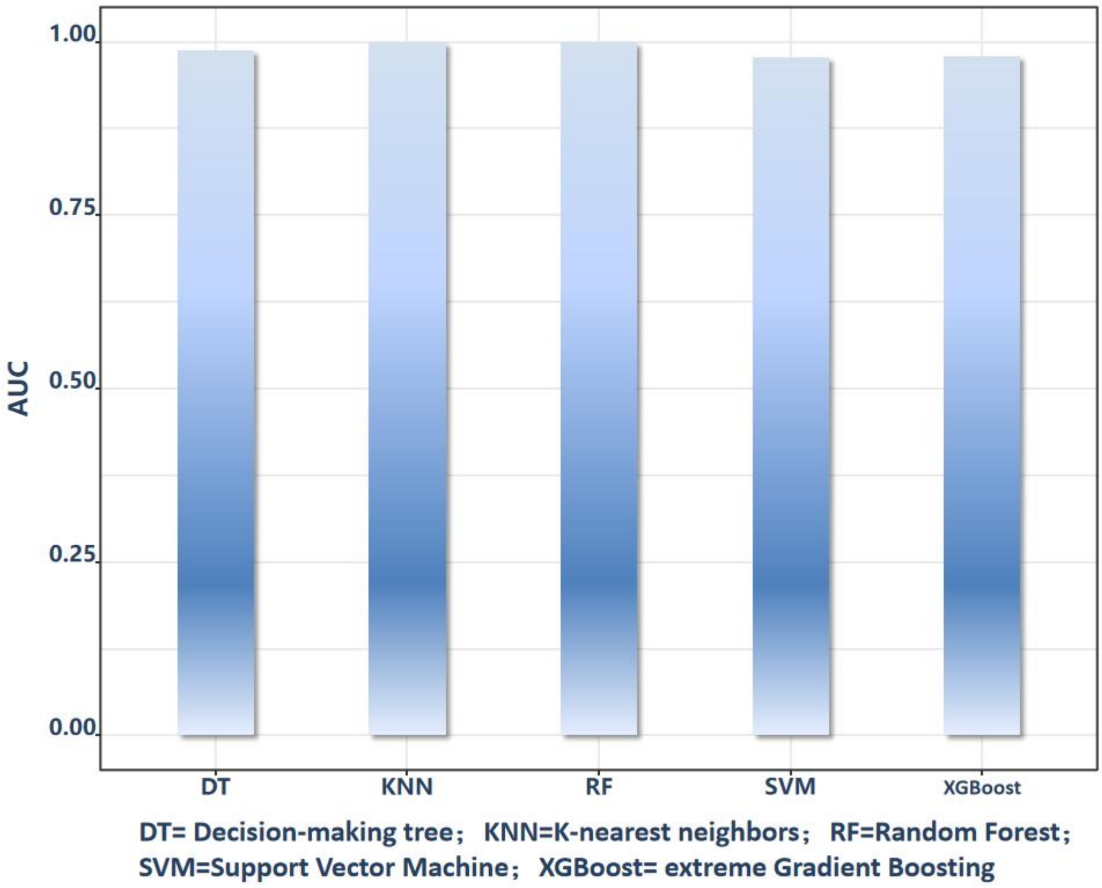

Figure S2. AUROCs for models in training set.

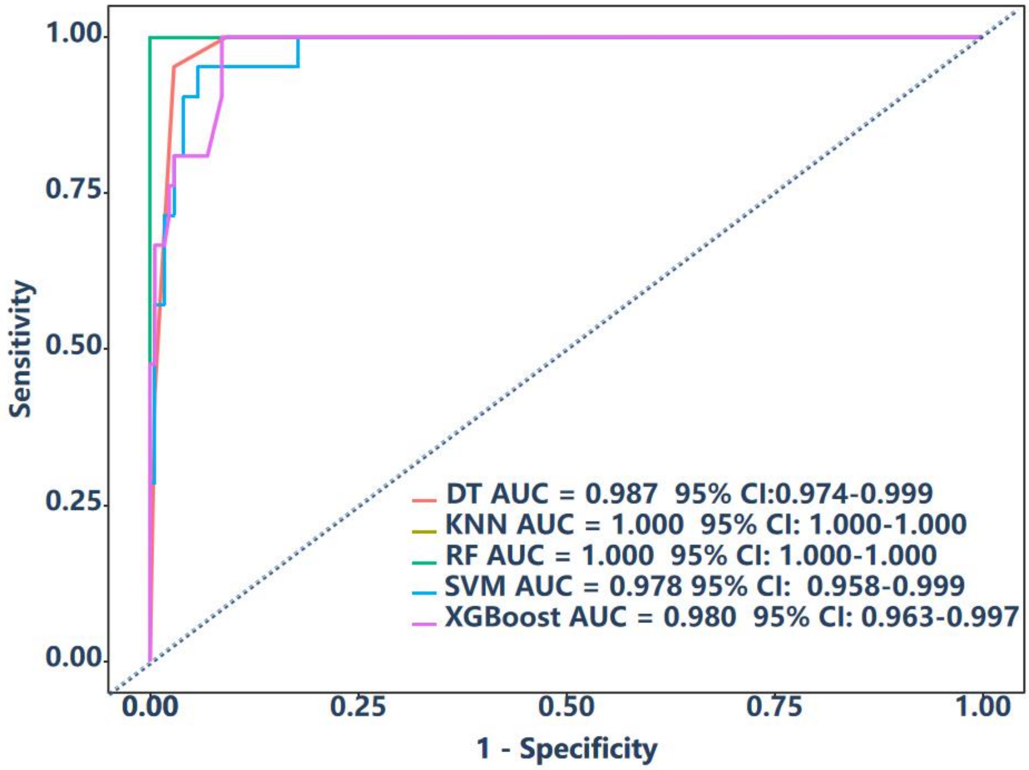

Figure S3. AUPRCs for models in training set.

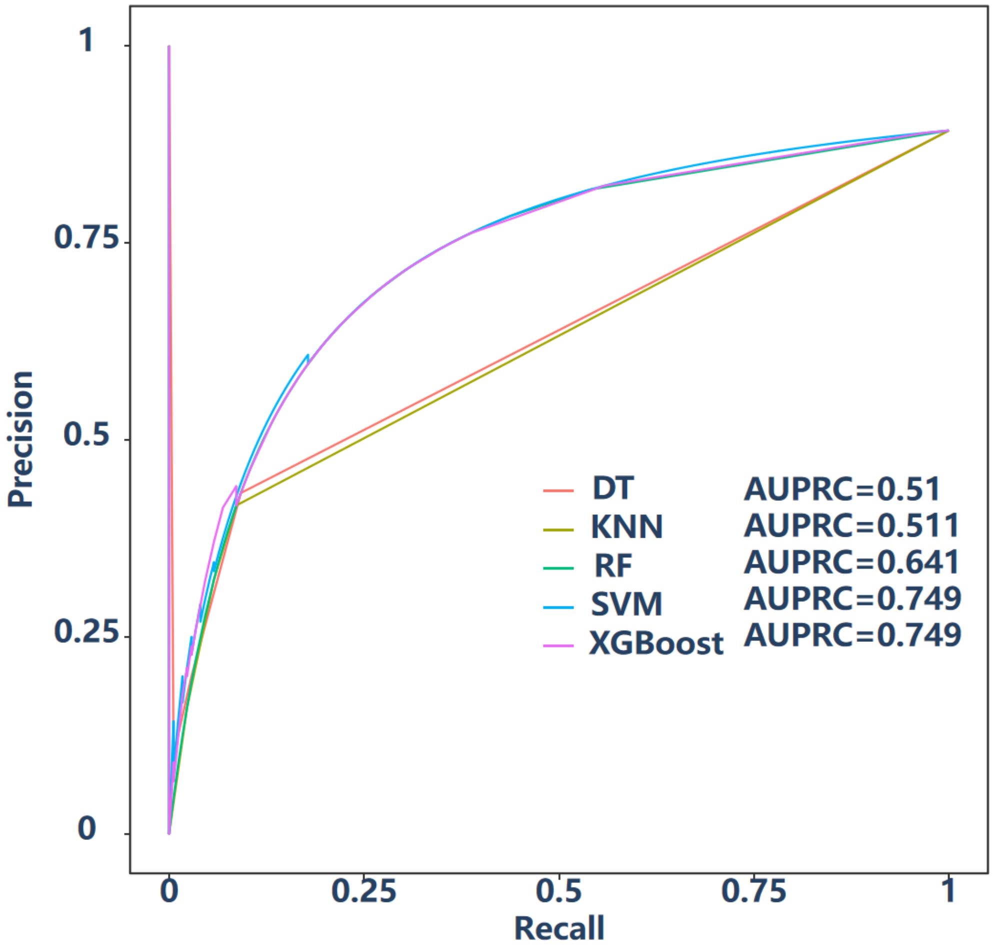

Figure S4. Specificity, sensitivity, F1 Score, recall, NPV and PPV for models in in training set.

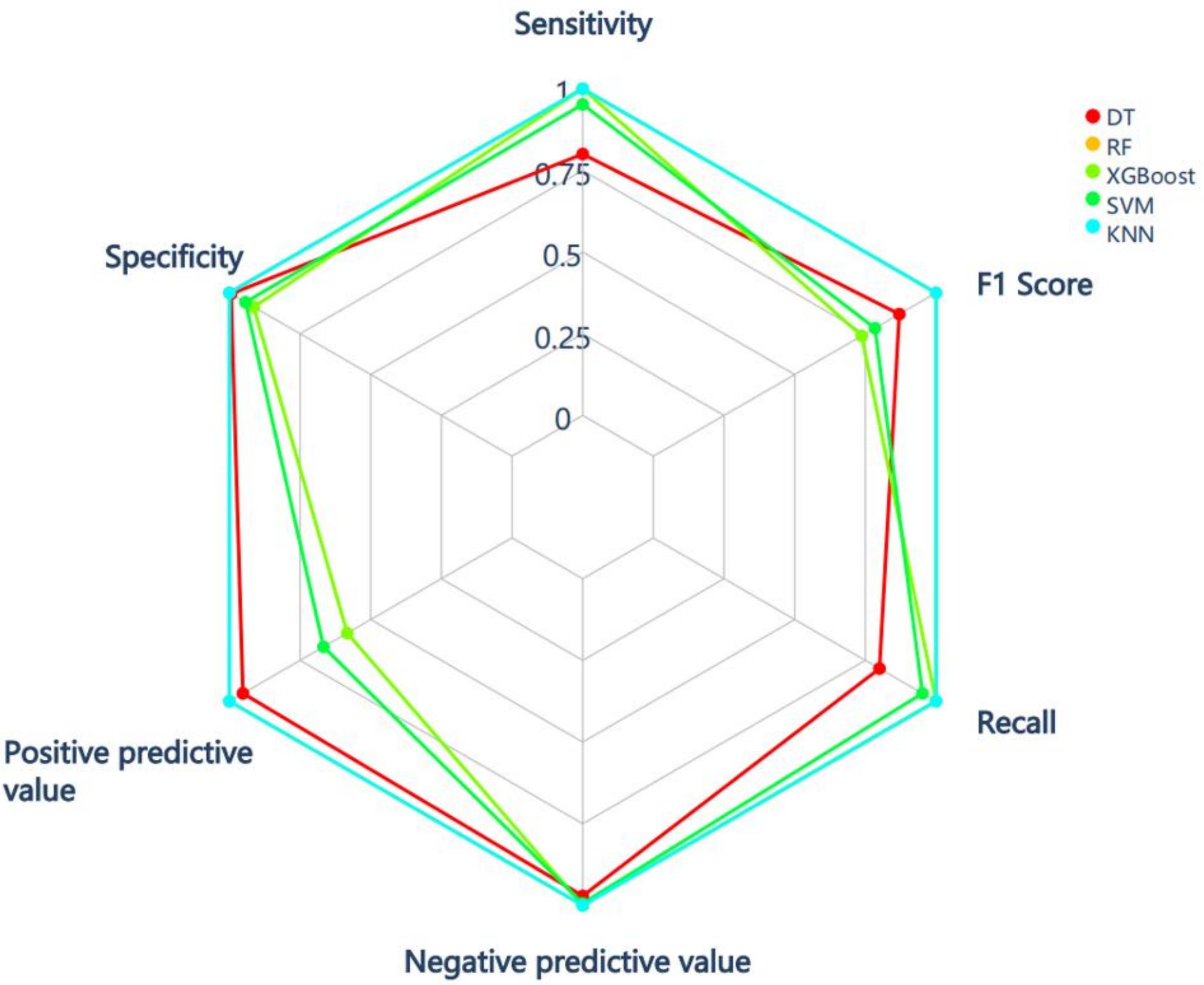

Figure S5. The area under the receiver operating characteristic curves for support vector machine in training and validation sets. AUC: area under the curve.

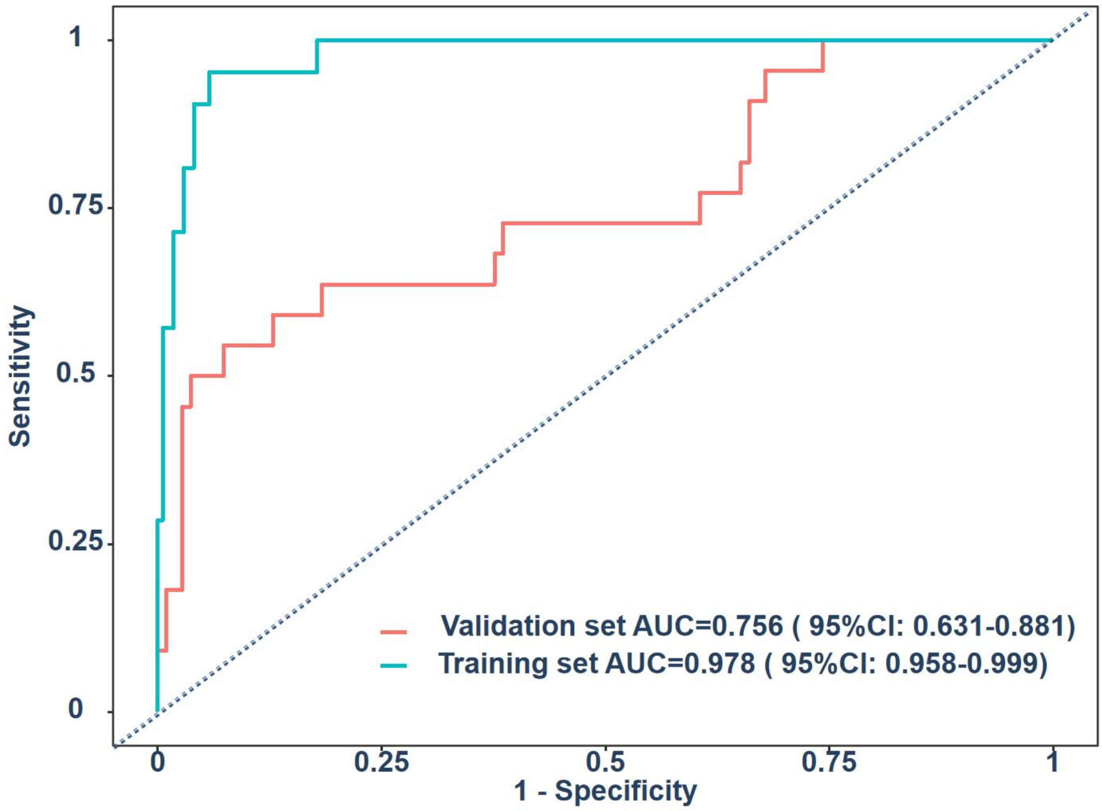

Figure S6. Confusion matrix plots for support vector machine in training and validation sets.

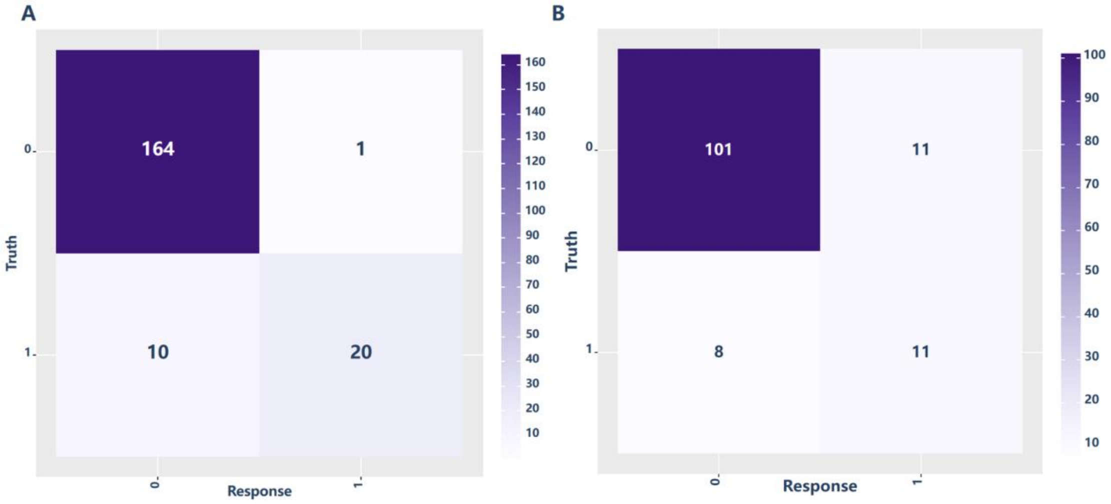

Figure S7. The final support vector machine (SVM) model, incorporating eight features, is readily applicable for clinical use in predicting major adverse cardiovascular and cerebrovascular events (MACCEs). By inputting actual values for these eight features, the application autonomously calculates and displays the probability of MACCEs occurrence. Additionally, the force plot for patients with aortic stenosis (AS) and heart failure with preserved ejection fraction (HFpEF) post-transcatheter aortic valve replacement (TAVR) elucidates the features influencing the prediction of "MACCEs." Specifically, the blue features on the right side of the plot indicate factors that drive the prediction towards the "non-MACCEs" classification, whereas the red features on the left side indicate factors that drive the prediction towards the "MACCEs" classification.

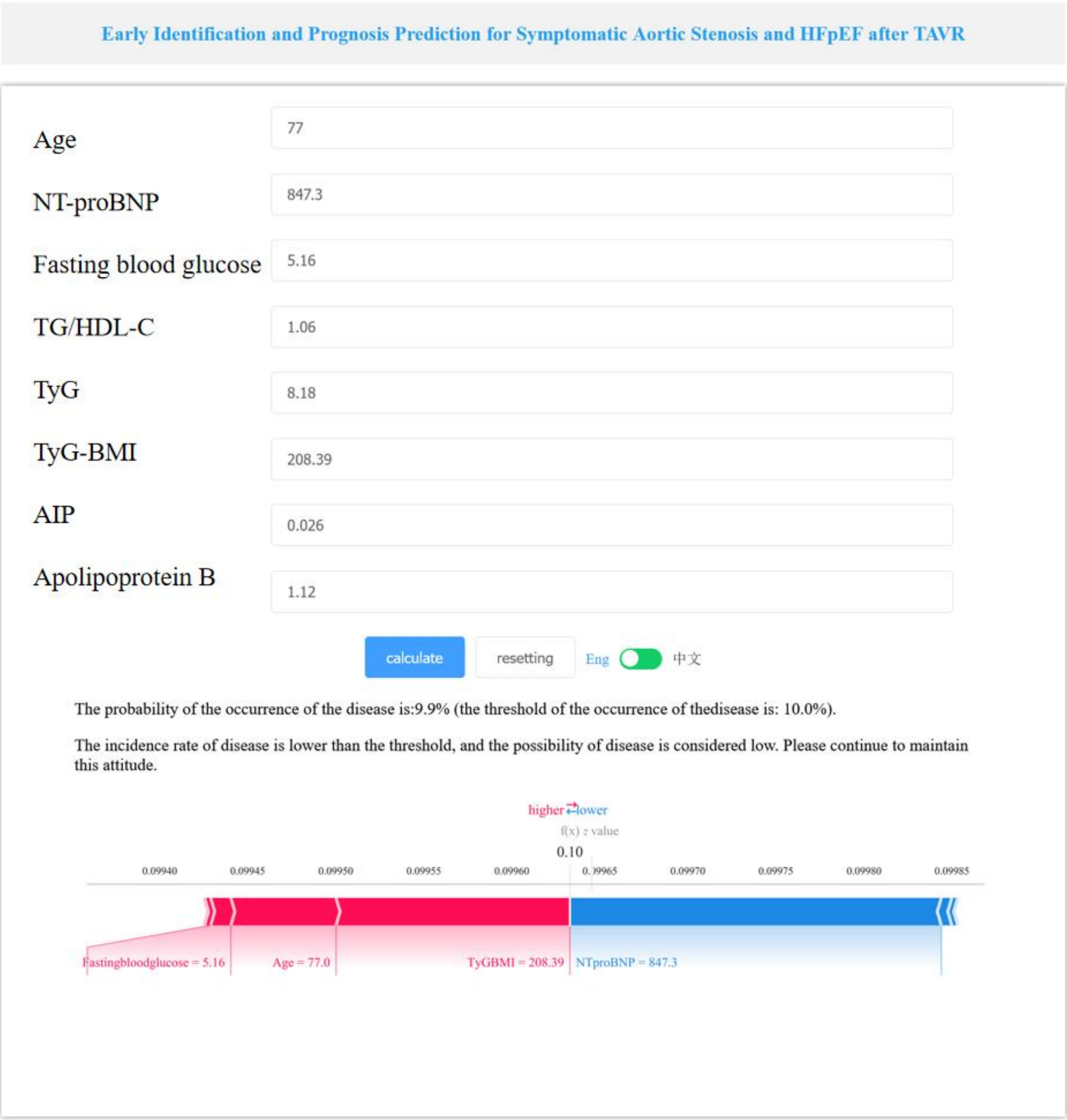

Supplementary material Methods

Complete-Case Analysis Strategy

Our study employed a complete-case analysis approach for managing missing data.

Variable Selection Criteria

We deliberately selected clinical variables that are routinely collected in standard TAVR evaluation protocols, prioritizing parameters with high availability across centers. The 58 initial variables included in our analysis were specifically chosen because they are consistently documented as part of standard clinical care for TAVR candidates.

Missing Data Assessment

Prior to cohort finalization, we conducted a comprehensive missing data audit. The selected biomarkers and clinical parameters demonstrated high completion rates (100% for core variables) across the participating centers.

Exclusion Protocol: Patients with missing values for any of the selected variables were excluded from the final analysis cohort. This resulted in the exclusion of 23 patients (4.5% of the initially eligible population).

This complete-case approach was preferred over imputation techniques for the following reasons: First, the low proportion of missing data (4.5%) minimizes potential selection bias. Second, utilizing a complete dataset enhances the reliability of our feature selection algorithms. Lastly, a model developed using complete data simplifies future clinical implementation by eliminating the need for imputation procedures.
